# Overexpression of a disintegrin and metalloproteinase 21 is associated with motility, metastasis, and poor prognosis in hepatocellular carcinoma

**DOI:** 10.1038/s41598-017-15800-z

**Published:** 2017-11-14

**Authors:** Hiroki Honda, Masaaki Takamura, Satoshi Yamagiwa, Takuya Genda, Ryoko Horigome, Naruhiro Kimura, Toru Setsu, Kentaro Tominaga, Hiroteru Kamimura, Yasunobu Matsuda, Toshifumi Wakai, Yutaka Aoyagi, Shuji Terai

**Affiliations:** 1 Division of Gastroenterology and Hepatology, Niigata University Graduate School of Medical and Dental Sciences, Niigata, Japan; 2 Department of Gastroenterology and Hepatology, Juntendo University Shizuoka Hospital, Shizuoka, Japan; 3 Division of Digestive and General Surgery Niigata University Graduate School of Medical and Dental Sciences, Niigata, Japan

## Abstract

Cell motility plays an important role in intrahepatic metastasis of hepatocellular carcinoma (HCC), and predicts poor prognosis in patients. The present study investigated the role of a disintegrin and metalloproteinases (ADAMs) in HCC, since these proteins are known to be associated with cell motility. We confirmed the expression of 12 ADAMs with putative metalloproteinase activity in HCC cells, and established a KYN-2 HCC cell line stably expressing short interfering RNA against ADAM21 to investigate the effect of ADAM21 deficiency on HCC cell motility and metastasis *in vitro* and *in vivo*. We also examined ADAM21 expression in a cohort of 119 HCC patients by immunohistochemistry. ADAM21 was overexpressed in KYN-2 cells, and its knockdown reduced invasion, migration, proliferation, and metastasis relative to controls. In clinical specimens, ADAM21 positivity was associated with vascular invasion, large tumor size, high histological grade, and lower overall and recurrence-free survival as compared to cases that were negative for ADAM21 expression. A multivariate analysis revealed that ADAM21 positivity was an independent risk factor for overall (P = 0.003) and recurrence-free (P = 0.001) survival. These results suggest that ADAM21 plays a role in HCC metastasis and can serve as a prognostic marker for disease progression.

## Introduction

Hepatocellular carcinoma (HCC) is a major cause of cancer-related mortality, with approximately 700,000 deaths worldwide and more than 30,000 in Japan each year^1,2^. Most HCC patients have poor prognosis due to the high incidence of intrahepatic metastasis and recurrence after curative therapeutic intervention^3^. The former results from the dispersal of cells around the tumor periphery to surrounding liver tissues through the portal vein^4,5^, although the underlying mechanisms are not well understood. We previously reported that HCC cell lines with high motility exhibit high rates of intrahepatic metastasis *in vivo* ^6^. Inhibiting motility in highly metastatic HCC cell lines by overexpressing a dominant-negative Rho kinase or by treatment with Rho kinase inhibitor decreased metastatic potential, indicating that cell motility plays an important role in intrahepatic metastasis of HCC^6,7^.

A disintegrin and metalloproteinases (ADAMs) were originally discovered as a family of proteins with sequence similarity to the reprolysin family of snake venomases^8^. To date, 21 members of the human ADAM family have been identified^9^. ADAMs share an N-terminal secretory signal sequence, prodomain, and metalloprotease, disintegrin-like, cysteine-rich, epidermal growth factor-like, transmembrane, and cytoplasmic domains^10^. More than half of ADAMs have an active site in the metalloprotease domain with a common HEXXHXXGXXH sequence, similar to matrix metalloproteinases^11^. Protease-type ADAMs are closely associated with cell invasion, proliferation and metastasis. Upregulation of ADAM17 is a pathological feature of HCC^12^; suppression of ADAM17 by the microRNA miR-122 inhibited *in vitro* migration, invasion, *in vivo* tumorigenesis, intrahepatic metastasis, and local invasion in the livers of nude mice^13^. However, there have been no systematic studies on the role of other protease-type ADAMs in HCC.

To address this issue, the present study analyzed the expression of 12 protease-type ADAMs in HCC cell lines and found that ADAM21 was overexpressed in the highly motile and metastatic KYN-2 HCC cell line. We also found that high ADAM21 expression predicted poor prognosis in HCC patients. These results provide the first evidence that ADAM21 plays an important role in the malignant progression of HCC.

## Results

### Real-time quantitative PCR analysis of proteinase-type ADAM expression in HCC cell lines

**Figure 1:**
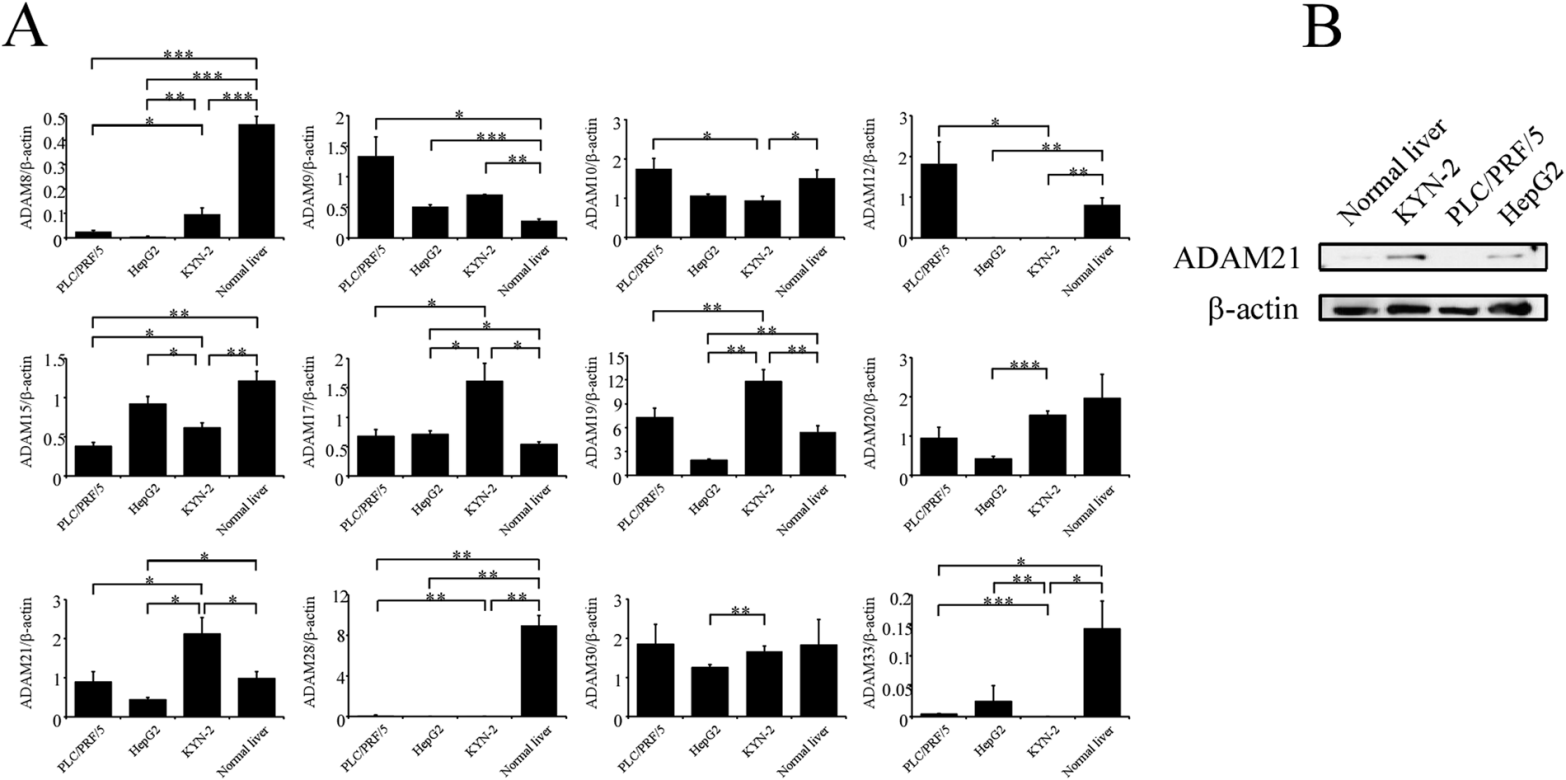
Expression of proteinase-type ADAMs in KYN-2, PLC/PRF/5, and HepG2 cells and normal liver tissue. (**A**) Relative mRNA levels of 12 ADAMs were analyzed by real-time PCR. Values were normalized to the level of β-actin in each sample. Experiments were performed three times. Data represent means ± SD. *P < 0.05, **P < 0.01, ***P < 0.001 (Student’s t test). (**B**) Immunoblot analysis of ADAM21 protein levels in HCC cell lines and human liver tissue lysate. β-actin served as a loading control.

We evaluated the mRNA levels of ADAM8, 9, 10, 12, 15, 17, 19, 20, 21, 28, 30, and 33—which have putative metalloproteinase activity—in KYN-2, PLC/PRF/5, and HepG2 cells and normal liver tissue by real-time PCR. ADAM17, 19, and 21 were more highly expressed in KYN-2 cells than in PLC/PRF/5 and HepG2 cells and normal liver (Fig. 1A). ADAM17 and 19 expression has been linked to many human cancers^9^, and ADAM17 is a target of the micro (mi)RNA miR-122 and is involved in HCC tumorigenesis and metastasis^13^. Many studies have reported that ADAM19 is a direct target of several miRNAs, including miR-153, -30c, or -145 and plays an important role in cell migration, invasion, and proliferation in various cancers^14–16^. Moreover, dysregulation of transforming growth factor-β/SMAD4 signaling leads to epigenetic silencing of its downstream target ADAM19 in ovarian cancer cells^17^. Therefore, in this study, we evaluated the expression and function of ADAM21 in HCC tumorigenesis and metastasis. An immunoblot analysis revealed that ADAM21 protein was highly expressed in KYN-2 cells and expressed at low levels in HepG2 cells and normal liver (Fig. 1B).

### ADAM21 knockdown inhibits HCC cell proliferation, migration, and invasion and induces cell-cycle arrest and apoptosis

**Figure 2:**
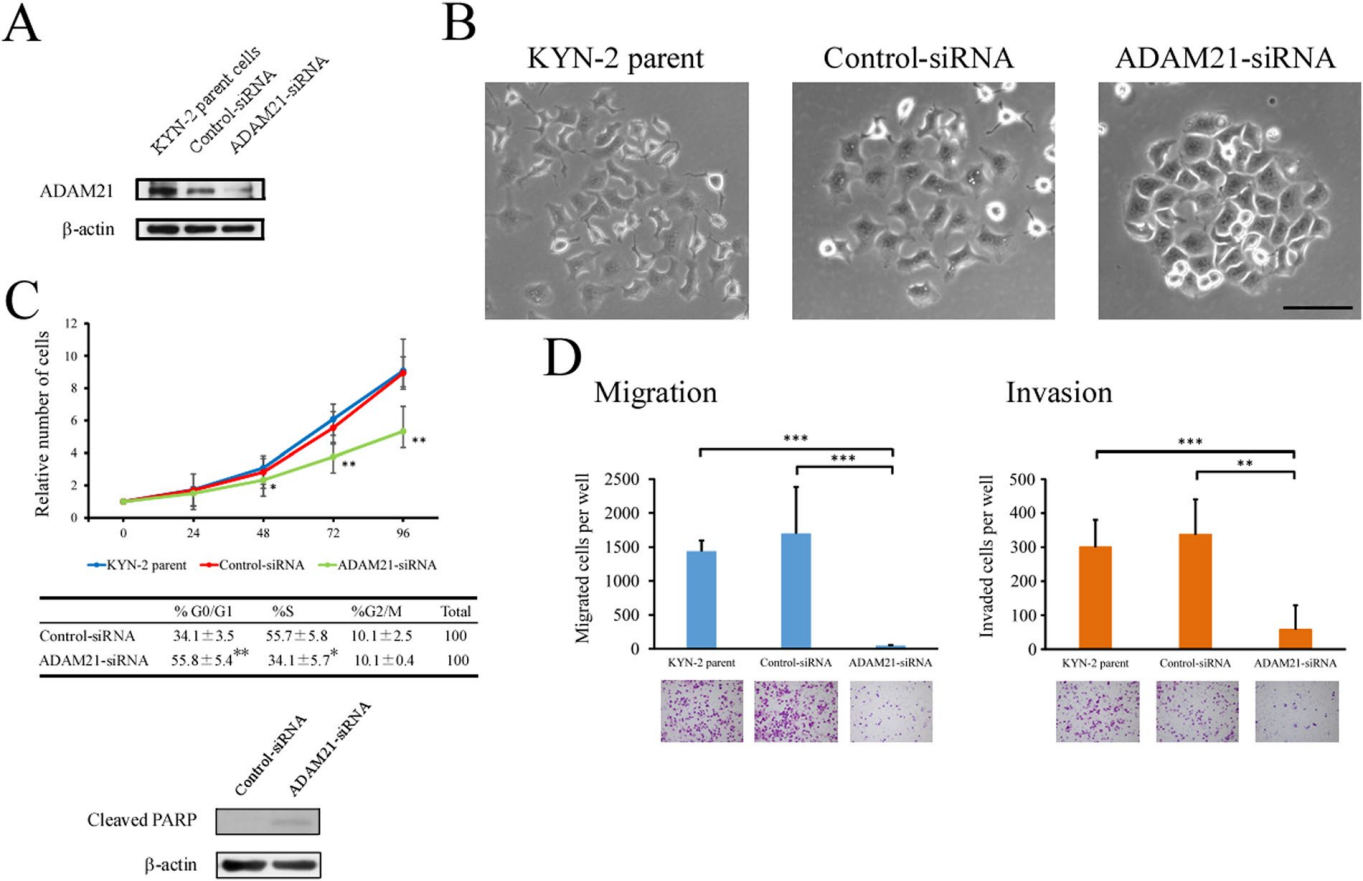
Effect of siRNA-mediated ADAM21 knockdown on cell morphology, proliferation, migration, and invasion *in vitro*. (**A**) Basal ADAM21 protein expression in KYN-2 parent cells or cells expressing control siRNA or siRNA against ADAM21, as detected by immunoblotting. (**B**) Phase-contrast micrographs of KYN-2 parent cells (left), or cells expressing control siRNA (middle) or an siRNA targeting ADAM21 (right). Bar = 100 μm. (**C**) (Upper panel) Proliferation of KYN-2 parent cells and cells expressing control siRNA or siRNA against ADAM21. The cell number (1.0 × 10^3^ cells) at 0 h was set as 1, and normalized measurements are presented as a fold increase. (Middle panel) Cell-cycle analysis at 96 h after seeding of KYN-2 cells expressing control siRNA or siRNA against ADAM21. (Lower panel) Immunoblot analysis of cleaved of poly (adenosine disphosphate)-ribose polymerase (PARP) 96 h after seeding KYN-2 cells expressing control siRNA or siRNA against ADAM21. β-Actin served as a loading control. *P < 0.05, **P < 0.01 vs. control siRNA-transfected cells. (**D**) Migration (left) and invasion (right) of KYN-2 parent cells and cells expressing control siRNA or siRNA against ADAM21. Data represent mean ± SD. **P < 0.01, ***P < 0.001 vs. KYN-2 parent cells or control siRNA-transfected cells.

To investigate the *in vitro* function of ADAM21, KYN-2 cells were stably transfected with either a control short interfering (si) RNA or one targeting ADAM21 (Fig. 2A). Similar to the parent cell line, cells expressing control siRNA had a ruffled membrane and fusiform shape without cell-to-cell contacts (Fig. 2B). In contrast, cell expressing the siRNA against ADAM21 had fewer membrane ruffles and formed relatively cohesive colonies (Fig. 2B). We investigated changes in the expression of epithelial–mesenchymal transition (EMT) markers such as E-cadherin, zona occludens (ZO)-1, N-cadherin, and vimentin by immunoblotting upon ADAM21 knockdown and found no changes in E-cadherin and ZO-1 levels, whereas N-cadherin and vimentin were not detected in KYN-2 cells expressing either control or ADAM21 siRNA (data not shown). The cell proliferation assay revealed that ADAM21 knockdown reduced the growth of KYN-2 cells (P < 0.05 at 48 and 72 h and P < 0.01 at 96 h vs. control siRNA-transfected cells) (Fig. 2C, upper panel). We performed a cell-cycle analysis to clarify the mechanism by which ADAM21 knockdown inhibits cell proliferation and found that 96 h after seeding, KYN-2 cells expressing ADAM21 siRNA were arrested in G0/G1, and a smaller proportion were in S phase as compared to KYN-2 cells expressing control siRNA (*P* < 0.01 and *P* = 0.01, respectively) (Fig. 2C, middle panel). Immunoblot analysis revealed detectable cleavage of poly (adenosine disphosphate)-ribose polymerase (PARP) (Fig. 2C, lower panel). In addition, migration and invasion assays revealed that ADAM21 siRNA-expressing KYN-2 cells showed significantly reduced migration (P < 0.001) and invasion (P < 0.01) (Fig. 2D). We also observed a similar effect in cells transfected with siRNA oligonucleotides (Supplemental Fig. 1A–C).

### ADAM21 knockdown slows tumor growth in a murine orthotopic implantation model

**Figure 3:**
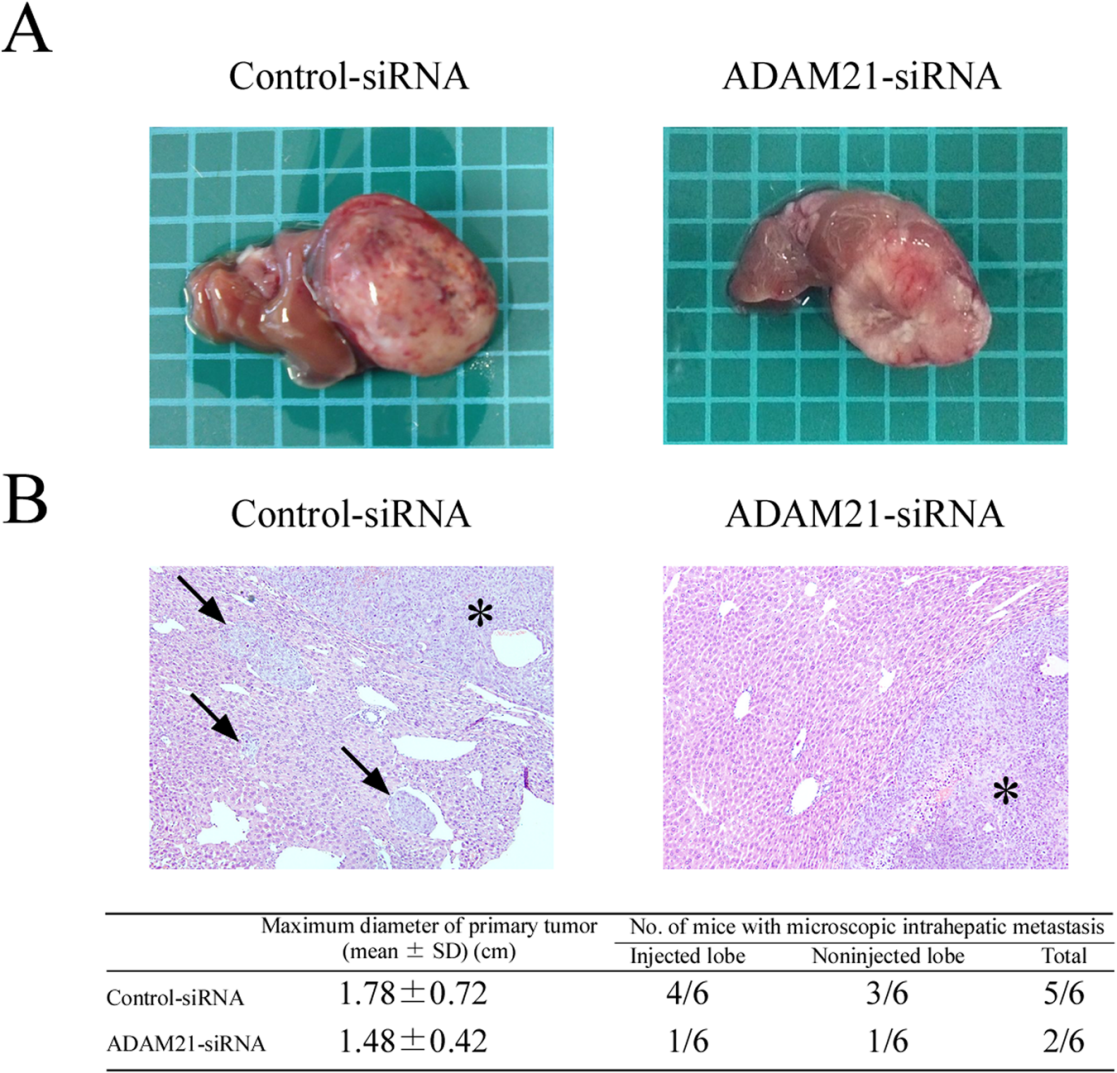
Effect of siRNA-mediated ADAM21 knockdown on HCC in a murine orthotopic implantation model. (**A**) Macroscopic view of KYN-2 cells expressing control siRNA or an siRNA against ADAM21 tumors. The square shows an area of 5 × 5 mm. (**B**) Histological analysis of tumors formed by KYN-2 cells expressing control siRNA (upper left) or an siRNA against ADAM21 (upper right). Asterisks indicate primary tumors developed in the injected lobe, and arrows indicate intrahepatic metastases (hematoxylin and eosin staining, original magnification: 100×). Hematogeneous intrahepatic metastasis of KYN-2 cells expressing control or ADAM21 siRNA (lower). Data represent the number of mice with hematogeneous intrahepatic metastasis divided by the total number of mice evaluated.

To determine whether ADAM21 is involved in tumorigenesis or intrahepatic metastasis *in vivo*, KYN-2 cells expressing siRNA against ADAM21 or control siRNA were orthotopically implanted into the livers of SCID mice. Tumors formed in all mice at the site of injection; however, the maximum diameter of the primary tumor was smaller in the ADAM21 siRNA group than in the control siRNA group (Fig. 3A); this corresponded to a lower incidence of intrahepatic metastasis in the former (Fig. 3B), although the difference was not statistically significant. These results indicate that ADAM21 knockdown may inhibit tumorigenesis and intrahepatic metastasis *in vivo*.

### High ADAM21 expression in HCC tumors is associated with clinicopathological parameters of HCC

**Figure 4:**
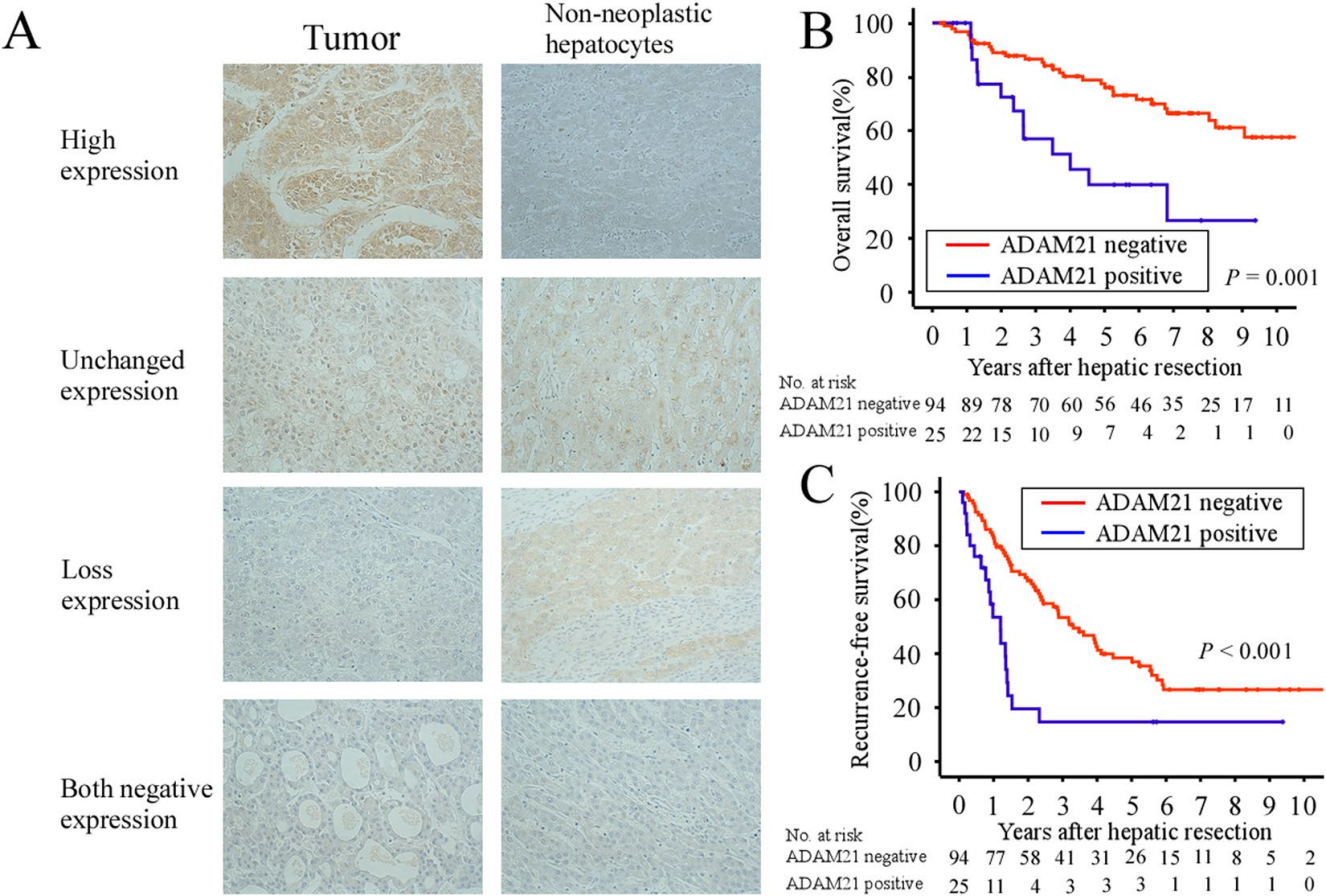
Immunohistochemical analysis of ADAM21 expression in 119 HCC tissue specimens and Kaplan-Meier survival curves for OS and RFS according to ADAM21 expression. (**A**) Representative images of HCC tissue specimens with various ADAM21 staining patterns (high expression, unchanged expression, loss of expression, or both negative expression). The final results were classified as ADAM21-positive (high expression) or ADAM21-negative (unchanged expression, loss expression, or both negative expression). Original magnification: 200×. (**B**,**C**) Post-resection survival (**B**) and RFS (**C**) were worse in ADAM21-positive patients (blue line) than in the ADAM21-negative group (red line; log-rank test, P = 0.001 and P < 0.001, respectively). The number of patients at risk reflects those remaining in the group at the indicated time point.

We examined ADAM21 protein expression in 119 patients with HCC by immunohistochemistry. ADAM21 protein was detected in the cytoplasm of tumor cells and exhibited various staining patterns (Fig. 4A). A total of 25/119 cases (21.0%) were positive for ADAM21 (high expression), whereas 94/119 (79.0%) were negative (unchanged expression, loss, or both negative expression). Table 1 summarizes the relationship between the degree of ADAM21 expression and various clinicopathological parameters; positive ADAM21 expression was related to large tumor size (P = 0.004), high histological grade (P = 0.014), and vascular invasion (P = 0.005).

**Table 1:** Relationship between clinicopathologic parameters and ADAM21 expression in patients with hepatocellular carcinoma.

Variable	Total (n = 119)	ADAM21		P value
		Positive (n = 25)	Negative (n = 94)	
Age (years)	70 (35–81)	71 (51–80)	70 (35–81)	0.396
Gender				
Male	85	17	68	0.804
Female	34	8	26	
Cirrhosis				
Absent	55	14	41	0.367
Present	64	11	53	
Serum AFP levels (ng/ml)				
≥20	56	14	42	0.371
<20	63	11	52	
Tumor size (cm)	3.1 (1.0–16.0)	3.8 (2.0–16.0)	3.0 (1.0–13.0)	0.004
Tumor multiplicity				
Solitary	73	14	59	0.645
Multiple	46	11	35	
Histological grade				
Well	26	1	25	0.014
Moderate/Poor	93	24	69	
Vascular invasion				
Absent	93	14	79	0.005
Present	26	11	15	
pTNM stage				
I + II	104	19	85	0.084
III	15	6	9	

### ADAM21 positivity is associated with poor overall survival (OS) after resection

A univariate analysis revealed that serum α-fetoprotein (AFP) level (P = 0.001), number of hepatic tumors (P = 0.009), vascular invasion (P = 0.010), and ADAM21 expression (P = 0.001) (Fig. 4B) were significant prognostic factors for overall survival (OS) (Table 2). Variables that were significant in the univariate analyses were subjected to multivariate analyses, which revealed that serum AFP levels ≥ 20 ng/mL (P = 0.009; hazard ratio [HR] = 2.398, 95% confidence interval [CI]: 1.250–4.601), multiple hepatic tumors (P = 0.036; HR = 1.932, 95% CI: 1.044–3.573), and ADAM21 positivity (P = 0.003; HR = 2.778, 95% CI: 1.414–5.458) were independent prognostic factors for unfavorable OS (Table 2).

**Table 2:** Factors significantly affecting overall survival after resection.

Variable	Modality	No. of patients	Overall survival		
			Univariate analysis	Multivariate analysis	P value
			P value*	Hazard ratio (95% CI)	
Age (years)	≥65	86	0.178		
	<65	33			
Gender	Male	85	0.103		
	Female	34			
Cirrhosis	Present	64	0.100		
	Absent	55			
Serum AFP levels (ng/mL)	≥20	56	0.001	2.398 (1.250–4.601)	0.009
	<20	63			
Tumor size (cm)	>5	18	0.967		
	≤5	101			
No. of hepatic tumors	Multiple	46	0.009	1.932 (1.044–3.573)	0.036
	Solitary	73			
Histological grade	Well	26	0.119		
	Moderate/Poor	93			
Vascular invasion	Present	26	0.010		
	Absent	93			
pTNM stage	I + II	104	0.119		
	III	15			
ADAM21 staining	Positive	25	0.001	2.778 (1.414–5.458)	0.003
	Negative	94			

*Log-rank test. *Abbreviations*: 95% CI, 95% confidence interval; AFP, alphafetoprotein; TNM, tumor-node-metastasis.

### ADAM21 positivity is associated with poor recurrence-free survival (RFS) after resection

A univariate analysis revealed that cirrhosis (P = 0.026), serum AFP levels (P = 0.003), number of hepatic tumors (P < 0.001), and ADAM21 positivity (P < 0.001) (Fig. 4C) were prognostic factors for recurrence-free survival (RFS) (Table 3). Multivariate analyses indicated that serum AFP levels ≥ 20 ng/mL (P = 0.007; HR = 1.854, 95% CI: 1.183–2.904), multiple hepatic tumors (P = 0.002; HR = 2.034, 95% CI: 1.293–3.200), and ADAM21 positivity (P = 0.001; HR = 2.473, 95% CI: 1.454–4.205) were independent prognostic factors for unfavorable RFS (Table 3).

**Table 3:** Factors significantly influencing recurrence-free survival after resection.

Variable	Modality	No. of patients	Recurrence-free survival		
			Univariate analysis	Multivariate analysis	P value
			P value*	Hazard ratio (95%CI)	
Age (years)	≥65	86	0.483		
	<65	33			
Gender	Male	85	0.312		
	Female	34			
Cirrhosis	Present	64	0.026		
	Absent	55			
Serum AFP levels (ng/mL)	≥20	56	0.003	1.854 (1.183–2.904)	0.007
	<20	63			
Tumor size (cm)	>5	18	0.341		
	≤5	101			
No. of hepatic tumors	Multiple	46	<0.001	2.034 (1.293–3.200)	0.002
	Solitary	73			
Histological grade	Well	26	0.205		
	Moderate/Poor	93			
Vascular invasion	Present	26	0.108		
	Absent	93			
pTNM stage	I + II	104	0.060		
	III	15			
ADAM21 staining	Positive	25	<0.001	2.473 (1.454–4.205)	0.001
	Negative	94			

*Log-rank test. *Abbreviations*: 95%CI, 95% confidence interval; AFP, alphafetoprotein; TNM, tumor-node-metastasis.

## Discussion

Recent advances in both diagnosis and therapeutics have allowed clinicians to identify HCC at earlier stages and recommend treatment in a timely manner. However, the prognosis of HCC remains poor, largely due to the high incidence of intrahepatic metastasis and recurrence after curative therapeutic intervention^3^. Additionally, although recent progress has advanced our knowledge and understanding of the molecular pathogenesis of HCC and the signaling pathways involved, the only mechanism-based pharmaceutical currently available is sorafenib^18^. There is therefore a need to identify biomarkers for predicting HCC recurrence and prognosis as well as new therapeutic targets.

We previously demonstrated that cell motility plays a critical role in intrahepatic metastasis of HCC^6,7^. In the present study, we examined protease-type ADAMs that are known to facilitate cell migration and invasion via proteolytic cleavage of extracellular matrix components, and found that ADAM21 was selectively overexpressed in KYN-2 cells. ADAM21 (also known as ADAM31) was first identified as a human testis-specific membrane metalloprotease with similarity to ADAM1 (fertilin-α), suggesting a role in sperm function^19^. In the developing and adult rodent central nervous system, ADAM21 is thought to be involved in axonal outgrowth and/or synapse formation^20^. There has been only one study on ADAM21 that identified this protein as a candidate factor involved in the invasion of HT29 colon cancer cells *in vitro* ^21^. Our findings support this earlier report and provide further evidence that silencing ADAM21 in KYN-2 cells causes cell-cycle arrest and inhibits cell proliferation, migration, invasion, and metastasis *in vitro* and *in vivo*. Although ADAM21 knockdown resulted in morphological changes to an epithelial phenotype, there were no changes in EMT marker expression, suggesting that ADAM21 knockdown alone may be not sufficient to reverse EMT. Recently, two definitive *in vivo* studies demonstrated that EMT is not relevant to metastasis but is associated with chemoresistance in breast and pancreatic cancers^22,23^. Further study is needed to determine whether this applies to intrahepatic metastasis of HCC. In our animal model, ADAM21 knockdown did not completely abolish intrahepatic metastasis, indicating that it is a complex process mediated by many factors^24^.

Our analysis of tissue samples from HCC patients undergoing curative resection showed that ADAM21 expression varied, and was present not only in tumor cells but also in adjacent non-neoplastic hepatocytes. It has been reported that many types of tumor produce growth factors that stimulate the proliferation and invasion of tumor cells themselves (in an autocrine fashion) and of surrounding normal cells (in a paracrine fashion)^25^. To our knowledge, there are no previous reports on the regulatory mechanism of ADAM21. One possible mechanism is the paracrine stimulation of adjacent non-neoplastic hepatocytes by unidentified factors that upregulate ADAM21 production by tumor cells.

Our clinicopathological study showed that high ADAM21 expression level in HCC tumors is closely related to large tumor size, high histological grade, and presence of vascular invasion and is an independent risk factor for poor OS and RFS. This is consistent with the results of our *in vitro* and *in vivo* studies and suggests that ADAM21 is a potential biomarker for HCC progression. However, further studies are needed in order to identify the physiological substrate(s) of ADAM21 and clarify its mechanism of action in HCC.

In conclusion, ADAM21-mediated cell motility plays an important role in HCC metastasis and is a prognostic biomarker for HCC patient OS and RFS after hepatectomy. These results indicate that therapeutic strategies targeting ADAM21 may be an effective treatment for preventing HCC metastasis and recurrence.

## Materials and Methods

### Cell lines and culture

The PLC/PRF/5 cell line was obtained from the Japanese Collection of Research Bioresources (Osaka, Japan); HepG2 cells were obtained from the RIKEN BioResource Center (Tsukuba, Japan); and the KYN-2 cell line was provided by Dr. H. Yano (Kurume University, Kurume, Japan)^26^. We previously characterized KYN-2 cells as a highly motile and metastatic HCC cell line, whereas PLC/PRF/5 and HepG2 cells exhibit low motility and metastatic potential^6^. All cells were cultured in Roswell Park Memorial Institute (RPMI) 1640 medium containing 10% fetal bovine serum (FBS), 100 U/ml penicillin, and 100 μg/ml streptomycin at 37 °C in a humidified atmosphere of 5% CO_2_. When required, the medium was supplemented with 0.25 μg/mL puromycin (Invitrogen, Carlsbad, CA, USA).

### Real-time PCR analysis

Total RNA was isolated using the RNeasy Mini kit (Qiagen, Valencia, CA, USA) according to the manufacturer’s protocol. cDNA was synthesized from 1 μg of total RNA using a PrimeScript RT reagent kit (Takara Bio, Otsu, Japan) and used as a template for real-time PCR on a Light Cycler System (Roche Diagnostics, Indianapolis, IN, USA). Primer/probe sets were selected from the Taqman Gene Expression Assays collection (Applied Biosystems, Foster City, CA) (Table 4). PCR conditions were as follows: 95 °C for 10 min, and 45 cycles of 95 °C for 10 s, 60 °C for 30 s, and 72 °C for 1 s. Results were normalized to the levels of human β-actin mRNA. Measurements were performed in triplicate.

**Table 4:** Genes and respective assay IDs for the predesigned TaqMan primer and probe sets.

Genes	Assay IDs
ADAM8	Hs00923280_m1
ADAM9	Hs00177638_m1
ADAM10	Hs00153853_m1
ADAM12	Hs01106104_m1
ADAM15	Hs00984794_m1
ADAM17	Hs00234221_m1
ADAM19	Hs00224960_m1
ADAM20	Hs01083178_s1
ADAM21	Hs01652548_s1
ADAM28	Hs00248020_m1
ADAM30	Hs00253969_s1
ADAM33	Hs00905552_m1
β-actin	Hs99999903_m1

### Antibodies

A mouse monoclonal anti-ADAM21 antibody was produced by MBL Co. (Nagoya, Japan) using a peptide corresponding to amino acids 710–722 as antigen. The antibody was used for immunoblotting (diluted 1:1000) and immunohistochemistry (diluted 1:100). A rabbit monoclonal anti-cleaved PARP (Asp214) antibody (Cell Signaling Technology, Beverly, MA) was used for immunoblotting (diluted 1:1000). A mouse monoclonal anti-β-actin antibody (clone AC-15; Sigma-Aldrich, St. Louis, MO) was used as a loading control for immunoblotting (diluted 1:5000).

### Immunoblotting

Cell lysates were prepared and immunoblotting was carried out as previously described^27^. Briefly, 30 μg of protein were resolved by sodium dodecyl sulfate polyacrylamide gel electrophoresis and transferred to an Immobilon membrane (Millipore, Bedford, MA, USA) that was blocked and probed with primary antibodies followed by the horseradish peroxidase (HRP)-conjugated secondary antibodies (GE Healthcare, Piscataway, NJ, USA). Peroxidase-labeled bands were visualized with enhanced chemiluminescence detection reagents (GE Healthcare) according to the manufacturer’s instructions. To confirm equal loading, the membrane was reprobed with anti-β-actin antibody.

### SiRNA-mediated knockdown of ADAM21

SiRNA against ADAM21 and control siRNA were generated using the piGENE vector (Nagase, Tokyo, Japan) and the following target sequences: ADAM21, GTAGAAACATTAGTACATC and control (T7STOP), CACCTTTTTTT. The siRNAs or control plasmid were transfected into KYN-2 cells with Lipofectamine 2000 (Invitrogen), and stable cell lines were generated from puromycin-resistant colonies.

### Cell proliferation assay

Cells (1.0 × 10^3^) resuspended in medium were seeded in a 96-well plate. Viable cells were quantified every 24 h with Cell Counting Kit-8 (Dojindo Laboratories, Kumamoto, Japan) according to the manufacturer’s protocol. Data represent mean ± SD of five independent experiments.

### Cell cycle analysis

Cell cycling was analyzed with the cell-clock assay (Biocolor, Carrickfergus, UK) according to the manufacturer’s protocol. Cell-clock dye is a redox dye that is readily taken up by live cells. In G0/G1, S, or G2/M phase, the dye is yellow, green, or dark blue in color, respectively. Cells in different cell cycle phases can be visualized from micrographs acquired in the bright field channel. The different fractions were quantified using ImageJ v.1.60 software (National Institutes of Health, Bethesda, MD, USA).

### Cell migration assay

Cell migration was evaluated with the transwell chamber assay (24-well-format with 8 μm pores; BD Biosciences, Bedford, MA, USA). Cells resuspended in RPMI 1640 medium containing 0.5% FBS were added to the upper chamber (5 × 10^4^ cells/well), and the lower chamber was filled with medium containing 10% FBS as a chemoattractant. After incubation for 24 h at 37 °C and 5% CO_2_, the membranes were fixed and stained with Diff-Quick solution (Sysmex International Reagents, Kobe, Japan). Cells that had migrated through the pores were counted under a light microscope. Samples were prepared in triplicate and the assay was repeated three times.

### Cell invasion assay

Cell invasion was assessed using BioCoat Matrigel Invasion Chambers (BD Biosciences) according to the manufacturer’s protocol. Cells resuspended in RPMI 1640 medium containing 0.5% FBS were added to the upper chamber (5 × 10^4^ cells/well), and the lower chamber was filled with medium containing 20% FBS as a chemoattractant. After incubation for 30 h at 37 °C and 5% CO_2_, the membranes were fixed and stained with Diff-Quick solution. Cells that had invaded through the pores were counted under a light microscope. Samples were prepared in triplicate and the assay was repeated three times.

### Orthotopic implantation in mice

Male homozygous C.B-17 SCID/SCID mice were purchased from CLEA Japan (Tokyo, Japan) and maintained at the Animal Center of Niigata University School of Medicine under specific pathogen-free conditions. *In vivo* experiments were carried out in full compliance with regulations and were approved by the Institutional Animal Care and Committee at the Niigata University (Niigata, Japan). Six-week-old mice were used for experiments. Orthotopic implantation of KYN-2 cells and KYN-2 transfectants was performed as previously described, with slight modification^6^. Briefly, cells were resuspended in phosphate-buffered saline containing 1% cold liquid Matrigel (Dow Corning, Corning, NY, USA; cat. no. 356230) at 1 × 10^8^ cells/ml and 20 μl of the cell suspension (2 × 10^6^ cells) were injected into the liver subserosa of anesthetized mice. Mice were sacrificed 4 weeks later and the livers were dissected, fixed in 10% formalin, and embedded in paraffin. To evaluate the frequency of intrahepatic metastasis, serial sections of liver tissue were stained with hematoxylin-eosin. Intrahepatic metastatic lesions were defined as either: (a) lesions in lobes other than the injected lobe; or (b) lesions that were clearly separate from the primary tumor.

### Patient population

A total of 119 consecutive patients with HCC underwent initial surgical resection with curative intent at Niigata University Medical and Dental Hospital from January 2001 to December 2008. There were 85 men and 34 women and the median age was 70 years (range: 35–81 years). None of the patients received preoperative chemo- or radiotherapy. Clinicopathologic factors were established according to general guidelines for primary liver cancer as defined by the Liver Cancer Study Group in Japan and the American Joint Committee on Cancer/International Union Against Cancer Tumor-Node-Metastasis (TNM) staging system^28,29^. Patients were divided into three groups according to TNM stage (I, II, and III); stage IV patients were excluded, as resection would most likely be non-curative. Written informed consent for the use of resected tissue samples was obtained from all patients in accordance with Declaration of Helsinki, and the study protocol was approved by the Human Ethics Review Committee of Niigata University Graduate School of Medical and Dental Sciences. All experiments were carried out in accordance with approved guidelines of Niigata University Graduate School of Medical and Dental Sciences.

### Patient follow-up after resection

Serum concentrations of AFP were measured, and abdominal ultrasonography and/or contrast-enhanced computed tomography was performed approximately 1 month after resection in all patients. Thereafter, patients were followed up every 3 months in outpatient clinics and monitored for disease recurrence by measuring serum AFP concentrations and/or by imaging. Patients were followed up until December 2014 with a median observation time of 63 months. At the time of disease status assessment, 42 patients had died from tumor recurrence and five had died of other causes with no evidence of disease; of the remaining patients, 38 were alive with recurrent disease and 34 were alive with no evidence of disease.

### Immunohistochemistry

Sections (5 μm thick) of formalin-fixed, paraffin-embedded tissue were deparaffinized in xylene and rehydrated in ethanol, treated with 0.3% hydrogen peroxide in methanol, and immersed in citrate buffer (10 mmol/L, pH 6.0). After autoclaving (121 °C, 10 min) and preincubating with normal swine serum, the sections were incubated overnight at 4 °C with primary antibody, which was detected with the Mouse/HRP Envision + system (Dako, Carpinteria, CA, USA). Immunoreactivity was visualized using 0.05% diaminobenzidine tetrahydrochloride solution containing 0.01% hydrogen peroxide. Sections were counterstained with hematoxylin. Leydig cells in the testis tissue served as a positive control for ADAM21; negative controls were prepared by substituting normal mouse serum for primary antibody and showed no detectable labeling.

ADAM21 expression in tumor specimens was evaluated by comparison with adjacent non-neoplastic hepatocytes and was classified as high expression, unchanged expression, or loss (i.e., signal intensity of the tumor specimen was higher than, similar to, or lower than that of non-neoplastic hepatocytes, respectively) or both negative expression (i.e., the tumor specimen and non-neoplastic hepatocytes both showed no signal). However, the final results were recorded only as negative (unchanged expression, loss, or both negative expression) or positive (high expression). Hematoxylin and eosin staining and immunohistochemical analyses were carried out by two independent observers (H.H. and N.K.) who were blinded to patients’ clinicopathologic information.

### Statistical analysis

Continuous variables are expressed as means ± standard deviation or median (range). Associations among variables were evaluated with the Student’s t test, Mann-Whitney U test, χ2 test, or Fisher’s exact test as appropriate. Medical records and survival data were obtained for all patients. Deaths from other causes were treated as uncensored cases. The Kaplan-Meier method was used to estimate the cumulative incidences of events, and differences in these incidences were analyzed with the log-rank test. Univariate and backward stepwise multivariate survival analyses were performed using the Cox proportional hazards model. Statistical analyses were performed using SPSS v.17.0 for Windows (SPSS Inc., Chicago, IL, USA). All tests were two-sided, and P values < 0.05 were considered statistically significant.

## Electronic supplementary material

Supplementary Information
