# Supplementary material for: Overexpression of a disintegrin and metalloproteinase 21 is associated with motility, metastasis, and poor prognosis in hepatocellular carcinoma
Source: Sci Rep. 2017 Nov 14;7:15485. doi: 10.1038/s41598-017-15800-z (PMC5686078; doi:10.1038/s41598-017-15800-z)
Supplement: Supplementary file 1 — Supplementary Information [file 41598_2017_15800_MOESM1_ESM.doc]

**Supplementary Information**

**Overexpression of a disintegrin and metalloproteinase 21 is associated with motility, metastasis, and poor prognosis in hepatocellular carcinoma**

Hiroki Honda^1^, Masaaki Takamura^1,*^, Satoshi Yamagiwa^1^, Takuya Genda^2^, Ryoko Horigome^1^, Naruhiro Kimura^1^, Toru Setsu^1^, Kentaro Tominaga^1^, Hiroteru Kamimura^1^, Yasunobu Matsuda^1^, Toshifumi Wakai^3^, Yutaka Aoyagi^1^, and Shuji Terai^1^

^1^Division of Gastroenterology and Hepatology, Niigata University Graduate School of Medical and Dental Sciences, Niigata, Japan, ^2^Department of Gastroenterology and Hepatology, Juntendo University Shizuoka Hospital, Shizuoka, Japan, ^3^Division of Digestive and General Surgery Niigata University Graduate School of Medical and Dental Sciences, Niigata, Japan

**Supplemental Materials and Methods**

***RNA interference***

An siRNA targeting ADAM21 (ON-TARGET *plus* SMART pool, L-004524) and negative control siRNA (ON-TARGET *plus* non-targeting pool, D-001810) were purchased from Dharmacon (Lafayette, CO, USA). Cells seeded in 6-well plates were transfected with 50 nM siRNA using Lipofectamine RNAiMAX Reagent (Invitrogen, Carlsbad, CA, USA) according to the manufacturer’s instructions. Cells were analyzed by immunoblotting 72 h later and were used for *in vitro* assays 48 h later.

**Supplemental Figure legends**

**Supplemental Figure 1.** Effect of ADAM21 knockdown using siRNA oligonucleotides on cell proliferation, migration, and invasion in vitro. (A) Immunoblot analysis of ADAM21 protein levels in KYN-2 cells expressing control siRNA or siRNA against ADAM21. β-Actin served as a loading control. (**B**) Proliferation of KYN-2 cells expressing control siRNA or siRNA against ADAM21. The cell number (1.0 × 10^3^ cells) at 0 h was set as 1, and normalised measurements are presented as a fold increase. **P < 0.001 vs. control siRNA-transfected cells. (**C**) Migration (left) and invasion (right) of KYN-2 cells expressing control siRNA or siRNA against ADAM21. Data represent mean ± SD. *P < 0.05, **P < 0.001 vs. KYN-2 control siRNA-transfected cells.

**Supplemental Figure 2.** Complete version of the immunoblot of the Figure 2C (lower panel). Immunoblot analysis of cleaved of poly (adenosine disphosphate)-ribose polymerase (PARP) 96 h after seeding KYN-2 cells expressing control siRNA or siRNA against ADAM21. The blot was stripped and reprobed for β-actin, which served as a loading control.

**Supplemental Figure 1.**

**
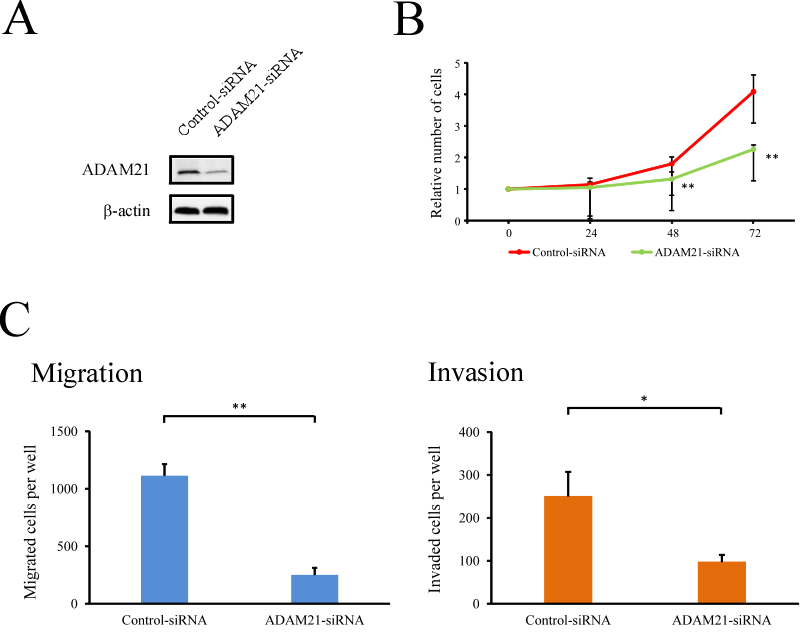
**

**Supplemental Figure 2.**

**
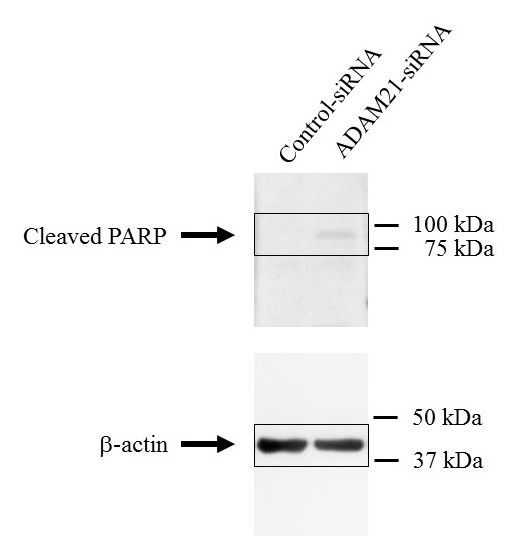
**
